# Dermoid Cyst Free in Abdomen during Pregnancy

**Published:** 1916-03

**Authors:** 


**Dermoid Cyst Free in Abdomen During Pregnancy.** Jasper Halpenny, Winnipeg, Can. Pract & Rev., Nov. 1915, reports the case of a woman aged about 35. In Nov. 1913, she had a 3-months miscarriage. Mch. 28. 1915, when about 4½ months pregnant, while working about the house, she felt a sudden sharp pain in the right side and felt faint and nauseated. 3 days later she was admitted to the hospital with a temperature of 100.8 and pulse of 72. White cells 12000. Operation was performed with the tentative diagnosis of acute inflammation of the appendix. A free, partly gangrenous mass, 2-3 the size of a fist, presented. This proved to a dermoid cyst whose pedicle had become twisted off, leaving practically no shreds at the stump. A chronically adherent appendix was removed but no exploration was made and the wound was closed without drainage. Uneventful recovery.

